# Polymeric Membrane Contactors for CO_2_ Separation: A Systematic Literature Analysis of the Impact of Absorbent Temperature

**DOI:** 10.3390/polym17101387

**Published:** 2025-05-18

**Authors:** Edoardo Magnone, Min Chang Shin, Jung Hoon Park

**Affiliations:** Department of Chemistry & Biochemical Engineering, Dongguk University, Manhae gwan, Room E629, 30, Pildong-ro 1gil, Jung-gu, Seoul 100-715, Republic of Korea; magnone.edoardo.korea@gmail.com (E.M.); gogokill31@naver.com (M.C.S.)

**Keywords:** separation, polymeric hollow fiber membranes, gas–liquid membrane contactors, CO_2_, liquid-phase temperature, physical absorption, chemical absorption

## Abstract

Global warming, driven significantly by carbon dioxide (CO_2_) emissions, necessitates immediate climate action. Consequently, CO_2_ capture is essential for mitigating carbon output from industrial and power generation processes. This study investigates the effect of absorbent temperature on CO_2_ separation performance using gas–liquid polymeric hollow fiber membrane (HFM) contactors. It summarizes the relationship between liquid-phase temperature and CO_2_ capture efficiency across various physical and chemical absorption processes. Twelve relevant studies (nine experimental, three mathematical), providing a comprehensive database of 104 individual measurements, were rigorously analyzed. Liquid-phase temperature significantly influences CO_2_ separation performance in HFM contactors. In particular, the present analysis reveals that, overall, for every 10 °C temperature increase, physical absorption performance decreases by approximately 3%, while chemical absorption performance improves by 3%, regardless of other parameters. This empirical law was confirmed by direct comparisons with additional experimental results. Strategies for further development of these processes are also proposed.

## 1. Introduction

### 1.1. Background

The removal and recovery of carbon dioxide (CO_2_), the most significant greenhouse gas, has attracted a lot of attention as a consequence of an increase in greenhouse gas emissions [1]. Typically, two distinct contacting processes—such as absorption and stripping towers—are used for CO_2_ capture and then for its extraction from absorbents. This is particularly important when considering the presence of CO_2_ as an undesirable side product in various industrial processes, where gas streams require treatment before being dispersed in the environment [2].

Gas–liquid polymeric membrane contactors (MCs) represent an advanced phase-contacting device for CO_2_ removal [3,4]. The principle of an MC is to facilitate contact between two distinct phases using a porous membrane, which acts as a physical barrier between a CO_2_-rich gas phase and a CO_2_-poor liquid phase [3,4]. This enables the liquid phase on one side of the porous membrane to selectively capture CO_2_ from the other side. As CO_2_ is absorbed by the liquid, it is removed from the gas mixture. Unlike traditional approaches like cryogenic processes and pressure swing absorption, membrane separation techniques offered by MC present several advantages [5]. These encompass decreased energy consumption, diminished operational costs, compact unit sizes, large contact surfaces, and the absence of moving parts. Consequently, the utilization of polymeric hollow fiber membrane (HFM) systems holds particular promise due to their extensive contact areas and inherent scalability [6,7,8,9].

For a more comprehensive review of the gas separation concepts, gas transport mechanism, and fabrication process of HFMs, we recommend the recent reviews by Li et al. [8], Li et al. [10], and Imtiaz et al. [11].

HFMs offer benefits compared to flat sheet membranes, including a large membrane surface area and a sturdy structure with high-density packing [12]. A single polymeric HFM typically comprises a membrane material that shapes a hollow core to form a small tubular structure measuring approximately 0.5 to 10 mm in diameter. Normally, polymeric HFMs consist of microporous regions with a three-dimensional porous structure. They often have a solid layer either inside or outside and may exhibit radial finger-like formations [13].

CO_2_ separation through absorption using HFMs can be divided into physical and chemical absorption [14]. Physical absorption involves CO_2_ dissolving into a solvent (i.e., H_2_O) without any chemical reaction taking place, while chemical absorption entails a reaction between CO_2_ and the absorbing solvent [9,15,16]. In detail, chemical absorption involves a chemical reaction between CO_2_ and the liquid phase. In contrast, the driving force for CO_2_ removal in a physical solvent is based on the solubility of CO_2_ [16].

Efficient CO_2_ separation is essential for many clean energy applications [4]. This study aims to evaluate all existing evidence regarding the relationship between liquid-phase temperature and CO_2_ capture efficiency. The focus is on various physical and chemical absorption processes employed by different gas–liquid polymeric HFM devices. To the best of our knowledge, this is the first analysis examining the impact of liquid-phase temperature on CO_2_ capture performance in polymeric HFM contactors.

### 1.2. CO_2_ Capture Using a Physical Absorption Process

When CO_2_ gas dissolves in water, carbonic acid ($H_{2}{\mathrm{CO}}_{3\left(\mathrm{aq}\right)}$) is formed, from which hydrogen ions ($H_{3}O^{+}$) dissociate, increasing the acidity of the liquid system through the following reactions:

(1) $${\mathrm{CO}}_{2\left(g\right)}\;↔{\mathrm{CO}}_{2\left(\mathrm{aq}\right)}$$

(2) $$H_{2}O_{\left(l\right)}+{\mathrm{CO}}_{2\left(g\right)}\;↔H_{2}{\mathrm{CO}}_{3\left(\mathrm{aq}\right)}$$

(3) $$H_{2}{{\mathrm{CO}}_{3}}_{\left(\mathrm{aq}\right)}+H_{2}O_{\left(l\right)}\;↔{{\mathrm{HCO}}_{3}^{-}}_{\left(\mathrm{aq}\right)}+{\;H}_{3}O^{+}$$

(4) $${{\mathrm{HCO}}_{3}^{-}}_{\left(\mathrm{aq}\right)}+{\;H}_{2}O_{\left(l\right)}↔{{\mathrm{CO}}_{3}^{2-}}_{\left(\mathrm{aq}\right)}+{\;H}_{3}O^{+}$$

where the enthalpy values (∆H) for Equations (1), (3), and (4) are approximately −20 kJ, 10 kJ, and 13 kJ, respectively [17].

Table 1 shows how CO_2_ solubility in water changes with temperature, ranging from 0 to 90 °C at atmospheric pressure [18]. CO_2_ solubility in water decreases significantly with rising water temperature, particularly between 0 and 20 °C [19,20]. In addition, CO_2_ physical solubility decreases with increasing temperature (0–90 °C [18]; 0–100 °C [20]) and increases with increasing pressure (0.49–9.87 atm [18]; 25–700 atm [20]). For example, Boributh et al. [21] observed a substantial decrease in CO_2_ removal rate, from approximately 9 × 10^−4^ mol/m^2^s to about 1.5 × 10^−4^ mol/m^2^s, as the water temperature increased from 5 °C to 85 °C. This demonstrates that CO_2_ solubility in water is highly sensitive to even small temperature changes. At 25 °C, it was 0.815 mol/mol, while at 30 °C (just 5 °C higher), it decreased to 0.706 mol/mol, as reported by Versteeg and Van Swaaij [22]. Consequently, optimal conditions for the physical CO_2_ removal process occur at low temperatures [16,23].

### 1.3. CO_2_ Capture Using a Chemical Absorption Process

The chemical mechanism, intuitively determined by the properties of the absorbents, involves the capture and removal of CO_2_ from the gas stream. Research primarily focuses on three types of chemical absorbents: aqueous alkaline solvents such as sodium hydroxide (NaOH) and potassium hydroxide (KOH), alkali salt solvents like sodium carbonate (Na_2_CO_3_) and potassium carbonate (K_2_CO_3_), and aqueous amine solvents.

#### 1.3.1. Aqueous Alkaline Solvents

To enhance the solubility of CO_2_ in water (see Equations (1)–(4)), promoting increased deprotonation of $H_{2}{\mathrm{CO}}_{3}$ via Equations (3) and (4) with basic substances like Group I metal hydroxides is essential. Consequently, the chemical absorption process using an aqueous alkali hydroxide can be summarized by the following reaction:

(5) $$2{\;\mathrm{NaOH}}_{\left(\mathrm{aq}\right)}+{\mathrm{CO}}_{2\left(g\right)}\;↔{\mathrm{Na}}_{2}{\mathrm{CO}}_{3\left(\mathrm{aq}\right)}+{\;H}_{2}O_{\left(l\right)}$$

The CO_2_ from the CO_2_-rich phase is absorbed through the polymeric HFM system by an alkaline NaOH solution to produce dissolved sodium carbonate [17,24]. The CO_2_ removal reactions with NaOH and KOH are strongly exothermic, with a ∆H° of −90 kJ and −49 kJ, respectively [17]. This indicates an inverse relationship between temperature and the chemical affinity between CO_2_ molecules and the absorbent. Increasing the liquid absorbent’s temperature accelerates the chemical reaction between hydroxide ions and CO_2_ gas, which improves the CO_2_ capture reaction [25].

Furthermore, as the absorbent temperature increases, the viscosity of the liquid absorbent decreases while diffusion increases, leading to an enhancement in the mass transfer rate. For example, according to Vazquez et al. [26], the viscosities of 1 mol/dm^3^ NaOH at 25 °C and 40 °C were 0.9281 mPa s and 0.6817 mPa s, respectively. In conclusion, increasing the temperature of an aqueous alkaline solvent also proves beneficial in reducing its viscosity.

#### 1.3.2. Alkali Salt Solvents

A second absorption system can be based on alkali salt solvents (e.g., Na_2_CO_3_, K_2_CO_3_, etc.) that primarily function through the CO_2_ chemical absorption with the formation of stable carbonate and bicarbonate complexes [27]. However, these solvents have some disadvantages that limit their use in polymeric HFM applications, like relatively slow reaction rates, low mass transfer rates, and high operating temperatures. While some references indicate compatibility with HFM materials, they do not specify which polymers were employed or their chemical–thermal stability characteristics [28,29,30]. An extensive review by Borhani et al. provides a state-of-the-art review of research on CO_2_ capture using potassium carbonate-based processes [31].

#### 1.3.3. Aqueous Amine Solvents

The improvement of CO_2_ absorption by the use of aqueous amine solvents, particularly monoethanolamine (MEA; HOCH_2_CH_2_NH_2_; CAS 141-43-5), is the main focus of recent research on CO_2_ capture [32]. The reaction between a liquid primary amine like MEA and CO_2_ in a chemical absorption process is presented as follows:

(6) $${\mathrm{CO}}_{2}+2{\mathrm{HOCH}}_{2}{\mathrm{CH}}_{2}{\mathrm{NH}}_{2}↔{\mathrm{HOCH}}_{2}{\mathrm{CH}}_{2}{\mathrm{NHCOO}}^{-}{\;\mathrm{HOCH}}_{2}{\mathrm{CH}}_{2}{\mathrm{NH}}_{3}^{+}$$

Instead, the reaction of secondary amines (i.e., DEA; HN(CH_2_CH_2_OH)_2_; CAS 111-42-2) for CO_2_ chemical absorption is presented as follows:

(7) $${\mathrm{CO}}_{2}+2\mathrm{HN}{\left({\mathrm{CH}}_{2}{\mathrm{CH}}_{2}\mathrm{OH}\right)}_{2}↔{\left({\mathrm{HOCH}}_{2}{\mathrm{CH}}_{2}\right)}_{2}{\mathrm{NCOO}}^{-}\;{\left({\mathrm{HOCH}}_{2}{\mathrm{CH}}_{2}\right)}_{2}{\mathrm{NH}}_{2}^{+}$$

(8) $${\left({\mathrm{HOCH}}_{2}{\mathrm{CH}}_{2}\right)}_{2}{\mathrm{NCOO}}^{-}\;{\left({\mathrm{HOCH}}_{2}{\mathrm{CH}}_{2}\right)}_{2}{\mathrm{NH}}_{2}^{+}+H_{2}O↔{\left({\mathrm{HOCH}}_{2}{\mathrm{CH}}_{2}\right)}_{2}{\mathrm{NH}}_{2}^{+}{\;\mathrm{HCO}}_{3}^{-}+{\left({\mathrm{HOCH}}_{2}{\mathrm{CH}}_{2}\right)}_{2}\mathrm{NH},$$

In these last two cases (Equations (7) and (8)), it can be observed that, theoretically, 1 mol of aqueous primary and secondary amines always reacts with 0.5 mol of CO_2_.

The reaction between aqueous tertiary amines (i.e., TEA; N(CH_2_CH_2_OH)_3_; CAS 102-71-6) and CO_2_ results in the formation of the bicarbonate ion (${\mathrm{HCO}}_{3}^{-}$). This chemical reaction can be represented as follows:

(9) $${\mathrm{CO}}_{2}+\;R^{\prime }R^{\prime \prime }R^{\prime \prime \prime }N\;+H_{2}O↔{\mathrm{HCO}}_{3}^{-}+{\;R^{\prime }R^{\prime \prime }R^{\prime \prime \prime }N\;}^{+}H$$

(10) $${\mathrm{CO}}_{2}+\;N{\left({\mathrm{CH}}_{2}{\mathrm{CH}}_{2}\mathrm{OH}\right)}_{3}+H_{2}O↔{\mathrm{HCO}}_{3}^{-}+{\left({\mathrm{HOCH}}_{2}{\mathrm{CH}}_{2}\right)}_{3}N^{+}H$$

The stoichiometric absorption capacity of CO_2_ for tertiary amines can reach 1.0 mol of CO_2_ per mole of amine. Tertiary amines react with CO_2_ at the lowest rates because the kinetics of bicarbonate production are typically slow [33].

Primary amines typically exhibit high CO_2_ absorption heats, ranging from −80 to −100 kJ/mole [34]. Secondary amines provide a balance, combining the faster reaction rates of primary amines with the lower reaction heats of tertiary amines, with their typical reaction heat falling between −70 kJ/mol and −90 kJ/mol [34]. Tertiary amines work differently; the reaction mechanism of tertiary amines involves promoting CO_2_ hydration through bicarbonate ion (${\mathrm{HCO}}_{3}^{-}$) formation [35]. This fact makes the absorption rate slower compared to the others. However, their absorption heat, ranging between −50 and −70 kJ/mole, makes them appealing for CO_2_ capture using amine-based liquids due to the potential for reduced energy use during regeneration [34]. Thus, their relatively low absorption heat results in less heat generation during the reaction, requiring less energy for regeneration and leading to a more energy-efficient process.

The liquid feed temperature significantly affects the physical and chemical absorption of CO_2_. This, in turn, influences the overall CO_2_ capture performance of microporous HFM systems. Essentially, the optimal temperature for CO_2_ capture depends on achieving a balance between different properties. Increasing the liquid feed temperature can improve the CO_2_ chemical absorption properties. However, higher temperatures can also lead to increased water evaporation from the absorbent solution, potentially blocking the HFM pores and thereby reducing CO_2_ diffusion. On the other side, as temperature rises, the CO_2_ back-pressure can also increase, reducing the driving force for CO_2_ removal into the CO_2_-poor liquid phase [4]. In addition, temperature is also a critical factor in thermal amine degradation and corrosion risk when the CO_2_ loading is elevated [36].

This study investigates the role of liquid-phase temperature in optimizing CO_2_ separation processes using gas–liquid polymeric HFM contactors.

### 1.4. Outline

This study comprehensively examines the impact of liquid feed temperature on CO_2_ absorption within various polymeric HFM designs by selecting core literature on the topic of CO_2_ separation. Building on existing research, the paper is divided into two main parts. The first section provides a foundational overview of the fundamental mechanisms of CO_2_ capture, encompassing both physical absorption (using water as a solvent) and chemical absorption (using alkaline, alkali salt, and amine solvents).

The second section of the present work examines the effect of liquid feed temperature on CO_2_ capture performance in polymeric HFM devices, comprehensively analyzing the collective behavior observed across the gathered experimental data from various selected research studies. 

Finally, the paper concludes with a summary of key findings and offers suggestions for future research directions. This work provides an overview of how liquid feed temperature affects CO_2_ separation in polymeric HFM devices, focusing on both physical and chemical absorption processes and examining the impact of temperature on each.

## 2. Methodology

To identify relevant literature, a comprehensive keyword search was conducted across three major academic databases (Google Scholar, Web of Science, and Scopus). The search focused on two key areas:

- CO_2_ capture utilizing polymeric HFM devices;
- the influence of liquid feed temperature on CO_2_ absorption performance.

The keyword search included terms such as “CO_2_”, “hollow fiber membrane(s)”, “polymeric hollow fiber(s)”, “separation”, “liquid”, “phase”, “feed”, and “temperature”. Approximately 5000 scientific papers were found in the first search.

To ensure the quality of the dataset, the initial search results were subsequently carefully screened. Studies were automatically excluded from the initial database based on the absence of essential chemical and physical information regarding HFM characteristics, device type, and polymer composition. Studies that did not investigate the effect of liquid feed temperature on CO_2_ removal performance were excluded. In particular, to provide a consistent dataset across different studies, the following criteria were used for the final selection:

1. HFM contactors: A candidate study must focus on polymeric HFM contactors for CO_2_ removal;
2. Data presentation: The selected study must also present CO_2_ separation efficiency (%) or CO_2_ absorption flux (mol/m^2^s) as a function of the liquid-phase temperature of a CO_2_-poor solution. These data need to be displayed graphically or reported in tabular form;
3. Additional information: The study must clearly report important information such as the nature of the polymeric HFM, its physical characteristics, and geometrical factors (e.g., geometry, number of fibers, HFM dimensions, etc.).

The final selection process resulted in a collection of relevant studies that will be comprehensively discussed in the following sections [21,29,37,38,39,40,41,42,43,44,45,46].

## 3. Results

To the best of our knowledge, this is the first analysis to specifically examine the relationship between liquid-phase temperature and CO_2_ absorption performance in polymeric HFM devices, considering both physical and chemical absorption processes. Ghobadi et al. observed that in the polytetrafluoroethylene (PTFE) all alkanolamine solvents exhibited increased flux and separation rates at higher temperatures. Ghobadi et al. [37] observed that with polytetrafluoroethylene (PTFE) HFM (liquid phase velocity = 0.105 m/s; gas phase velocity = 0.421 m/s; absorbent concentration = 1.0 M; feed gas = 2.5/97.5 CO_2_/CH_4_), all alkanolamine solvents showed increased flux and separation rates at higher temperatures in both counter-current and co-current flow patterns. However, increasing the water temperature hinders the flux and separation efficiency of CO_2_ due to decreased CO_2_ solubility and increased water evaporation.

Mansourizadeh et al. [38] found that CO_2_ flux through polyvinylidene fluoride (PVDF) HFM significantly increases with increasing temperature in chemical absorbents. In contrast, the CO_2_ flux decreases with the physical absorption temperature due to reduced CO_2_ solubility and capillary condensation in the membrane pores. In a counter-current flow system (liquid flow rate = 120 mL/min; gas flow rate = 200 mL/min; absorbent concentration = 1.0–0.2 M; pressure liquid–pressure gas = 0.2 × 10^5^ Pa), Mansourizadeh [39] reported that CO_2_ stripping flux via PVDF HFM is significantly influenced by liquid-phase temperature. Increased temperature and decreased pressure both contribute to reduced CO_2_ solubility, thereby impacting the stripping flux. Atchariyawut et al. [40] observed that increasing the temperature of a NaOH absorbent enhances the CO_2_ flux due to faster reaction kinetics in a counter-current flow membrane module (liquid phase velocity = 1.31 m/s; gas flow rate = 200 mL/min; feed gas = 20/80 CO_2_/CH_4_). On the other hand, when using water as the absorbent, the CO_2_ flux decreases with increasing temperature due to reduced gas solubility.

In a polypropylene (PP) HFM (counter-current flow pattern; gas flow rate = 16 L/h; CO_2_ inlet concentration = 40 wt.%), Golkhar et al. [41] observed a decrease in CO_2_ removal efficiency as the liquid temperature increased from 30 °C to 70 °C. This was attributed to a significant decrease in CO_2_ solubility in water and increased water evaporation. Mohammaddoost et al. [42] observed a decrease in CO_2_ absorption flux with increasing liquid temperature, which they attributed to increased membrane resistance caused by vaporization in PP HFMs in a co-current flow pattern (liquid flow rate = 0.4 L/min; gas velocity = 5.0 L/min; feed gas = 40%vol CO_2_). Rahim et al. [43] demonstrated that the effect of temperature on CO_2_ removal flux through PVDF HFM (counter-current flow pattern; liquid flow rate = 25 mL/min; gas flow rate = 100 cm^3^/min; absorbent concentration = 0.5 M; feed gas = 10/90 CO_2_/CH_4_) is a complex interplay of factors, including solubility, chemical reaction kinetics, diffusion, and absorbent evaporation. Unlike MEA and NaOH, CO_2_ removal for amino acid salt solutions exhibits a less pronounced temperature effect due to a balance between enhanced reaction rates and reduced solubility.

Cao et al. [44] observed an initial linear increase in terms of CO_2_ absorption flux by using a PTFE HFM device in a counter-current flow design (liquid phase velocity = 0.012 m/s; gas phase velocity = 0.239 m/s; CO_2_ partial pressure = 15 kPa) with increasing liquid temperature, followed by a period of slower flux growth. While higher temperatures can enhance CO_2_ diffusion and reaction rates, they must be carefully considered to avoid adverse thermodynamic effects such as increased solvent volatility and reduced CO_2_ solubility. As highlighted by Yan et al. [45], increasing absorbent concentration enhances mass transfer rates due to increased CO_2_ solubility. However, excessively high concentrations can lead to membrane-wetting issues when using PP HFMs (counter-current flow design; liquid flow rate = 0.0503 m/s; gas flow rate = 0.211 m/s; feed gas = 14/86 CO_2_/N_2_+O_2_). Therefore, optimizing absorbent concentration is crucial to maximize performance while avoiding operational challenges.

Collectively, the studies reviewed above [37,38,39,40,41,42,43,44,45] provide valuable insights, as a comprehensive analysis of the impact of liquid feed temperature on CO_2_ removal properties across various polymeric HFM devices has, to the best of our knowledge, been lacking in the literature. As outlined in the Introduction, this work aims to address this gap by analyzing existing literature, integrating findings from different studies, and exploring the overall influence of liquid temperature on both physical and chemical absorption processes within HFM systems.

The present analysis focuses exclusively on traditional absorbers, deliberately excluding innovative absorbents such as ionic liquids [47]. This decision was made for several reasons. Firstly, the temperature–performance relationship for these novel materials remains uncertain. As noted by Faisal Elmobarak et al. [48], the relative importance of chemical breakdown within the ionic liquid (specifically, at the cation–anion interface) under varying operating temperatures is not fully understood. Secondly, a more extensive body of research and data is available for the selected traditional absorbers, enabling a more comprehensive analysis of the influence of temperature on CO_2_ separation performance. Finally, our focus on traditional absorbers ensures the immediate applicability of this study to current industrial CO_2_ separation processes.

Selected papers report on nine different experimental studies [37,38,39,40,41,42,43,44,45] and three modeling studies [21,29,46], with a total of 104 data points. Data points are distributed as follows: water (H_2_O; 37 points) [37,38,39,40,41,42], sodium hydroxide (NaOH; 13 points) [38,40,43], *N*,*N*-dimethylethanolamine (DMEA; 5 points) [44], methyl diethanolamine (MDEA; 5 points) [45], diethanolamine (DEA; 3 points) [37], and triethanolamine (TEA; 3 points) [37]. Finally, 22 data points are extrapolated according to Refs. [21,29,46]. It is noteworthy that only two studies employed a co-current flow HFM device [41,42] to minimize the possibility of partial membrane wetting (as reported in [41]). In all other references (see Refs. [37,38,39,40,43,44]), a counter-current flow configuration was used for the gas and liquid absorbent streams.

Table 2 summarizes the collected data on CO_2_ removal by physical absorption using water in a liquid absorption process with a gas–liquid polymeric HFM device [37,38,39,40,41,42]. The table includes information on HFM polymer type, liquid temperature range (T), number of polymeric fibers (Nf) used in the HFM device, HFM outer diameter (F.Od), HFM inert diameter (F.Id), HFM length (F.l), HFM pore diameter (M.Pd), HFM porosity (M.P), HFM bubble point (B.p), module outer diameter (M.Od), module inert diameter (M.Id), module length (M.l), contact area (C.A.), and packing density (P.D.%). Table 2 is organized by the Nf in the polymeric HFM contactor.

The selected HFM devices employed a range of polymeric fibers (Nf) from a single unit (Nf = 1) [37] up to Nf = 1200 [42]. These devices mainly used commercially available HFMs [37,40,41,42] for physical CO_2_ capture in gas–liquid membrane systems, investigating the effect of liquid-phase temperature. In contrast to commercially available options [37,40,41,42], other studies fabricated the polymeric HFMs themselves using a wet phase-inversion process [38,39]. The analysis of polymeric HFM contactors for physical CO_2_ capture revealed a mean fiber outer diameter (F.Od) of 1.19 mm, with a standard error (SE) of 0.33 mm. The mean fiber inner diameter (F.Id) for this category was 0.87 mm, accompanied by a SE of 0.29 mm. These data suggest that the fibers utilized for physical capture exhibit reasonably comparable and overall small dimensions. As expected, it can be observed that increasing the number of fibers per device results in a practically straight increase in the contact area (cm^2^).

The investigated liquid-phase temperature ranges varied depending on the polymeric HFM material:

- Polytetrafluoroethylene (PTFE) HFM: from 20 to 60 °C (ΔT = 40 °C) [37];
- Polyvinylidene fluoride (PVDF) HFM: from 10 to 40 °C (ΔT = 30 °C) [38,39];
- Polyvinylidene fluoride (PVDF) HFM: from 30 to 60 °C (ΔT = 30 °C) [40];
- Polypropylene (PP) HFM: from 30 to 70 °C (ΔT = 40 °C) [41];
- Polypropylene (PP) HFM: from 20 to 40 °C (ΔT = 20 °C) [42].

Figure S1a in the Supplementary Materials illustrates the ranges of liquid-phase temperatures investigated in the selected literature for physical CO_2_ capture using polymeric HFM contactors [37,38,39,40,41,42].

Table 3 summarizes key operational parameters for CO_2_ removal via chemical absorption in polymeric HFM-based liquid absorption processes [37,38,40,43,44,45].

The number of polymeric fibers (Nf) in the HFM devices used for chemical absorption studies ranged from 1 (PTFE HFM; MEA) [37] to 7000 (PP HFM; MDEA) [45]. The collected studies primarily employed PVDF HFMs [38,40,43], PTFE HFM [37,44], and PP HFMs [45]. The investigated liquid-phase temperature ranges were:

- Polytetrafluoroethylene (PTFE) HFM: from 20 to 60 °C (ΔT = 40 °C) [37];
- Polyvinylidene fluoride (PVDF) HFM: from 10 to 40 °C (ΔT = 30 °C) [38];
- Polyvinylidene fluoride (PVDF) HFM: from 30 to 60 °C (ΔT = 30 °C) [40];
- Polyvinylidene fluoride (PVDF) HFM: from 20 to 60 °C (ΔT = 40 °C) [43];
- Polytetrafluoroethylene (PTFE) HFM: from 25 to 40 °C (ΔT = 15 °C) [44];
- Polypropylene (PP) HFM: from 30 to 50 °C (ΔT = 20 °C) [45].

Figure S1b in the Supplementary Materials illustrates the ranges of liquid-phase temperatures investigated for chemical CO_2_ removal using polymeric HFM devices [37,38,40,43,44,45]. MEA [37,43,45] and NaOH [38,40,43] were the most frequently investigated chemical absorbents, representing over 64% of the data points in the collected literature. The remaining data points involve DEA [37], TEA [37], DMEA [37], and MDEA [45].

For chemical CO_2_ capture, the mean F.Od was 1.90 mm with a SE of 0.4 mm, and the mean F.Id was 1.41 mm with a SE of 0.37 mm. This suggests a reasonable uniformity in the dimensions of the polymeric HFMs utilized across different laboratories.

Overall, the dataset includes a balanced representation of data from physical absorption processes (35.5%), chemical absorption processes (43.3%), and modeling studies (21.2%). In conclusion, this comprehensive analysis includes 104 data points from experimental and modeling studies, providing a robust foundation for understanding the overall influence of liquid temperature on CO_2_ capture using polymeric HFM devices. The dataset encompasses a diverse range of polymers, absorbents, fiber configurations, and liquid-phase temperature ranges.

## 4. Discussion

All the selected experimental studies [37,38,39,40,41,42,43,44,45] clearly defined their objectives. Nearly all studies [37,38,39,40,41,42,43,44,45] utilized various polymeric HFMs with either physical or chemical absorbents to investigate the influence of key operating parameters on CO_2_ removal. These parameters included:

- Absorbent pressure (Refs. [38,39]);
- Absorbent concentration (Refs. [40,41,44,45]);
- Liquid flow rate (Refs. [37,38,39,40,41,44,45]);
- Gas flow rate (Refs. [37,39,40,41,44,45]);
- CO_2_ concentration in flue gas (Refs. [41,44,45]);
- Flow pattern (co-current and counter-current) (Ref. [37]);
- Packing density (Ref. [37]);
- pH solution (Ref. [43]).

For a meaningful comparison, the present study focuses solely on the effects of liquid-phase temperature on CO_2_ separation. The collected literature [21,29,37,38,39,40,41,42,43,44,45,46] can be categorized into three homogeneous groups:

1. Studies reporting CO_2_ separation efficiency as a percentage of CO_2_ absorbed (%) [37,41];
2. Studies presenting experimental results in terms of CO_2_ absorption flux (mol/m^2^s) [38,39,40,42,43,44,45].

The CO_2_ separation performance (μ) through an HFM device is defined in terms of CO_2_ removal efficiency in percent (%) in Refs. [41,45], using the following equation:

(11) $$\mu =\frac{\left(Q_{\mathrm{in}}\times C_{\mathrm{in}}-{\;Q}_{\mathrm{out}}\times C_{\mathrm{out}}\right)}{Q_{\mathrm{in}}\times C_{\mathrm{in}}}\times 100$$

where Q_in_ and Q_out_ denote the inlet and outlet gas flow rates (m^3^/h) for the HFM module, respectively. Similarly, C_in_ and C_out_ represent the inlet and outlet CO_2_ volumetric concentrations in the gas phase, respectively [41,45]. In most cases [37,40,41,43,44,45], the CO_2_ absorption flux ($J_{CO_{2}}$) across the HFMs was estimated using the following equation:

(12) $$J_{{\mathrm{CO}}_{2}}=\frac{\left(Q_{\mathrm{in}}\times C_{\mathrm{in}}-{\;Q}_{\mathrm{out}}\times C_{\mathrm{out}}\right)\times 273.15\times 1000}{22.4\times T_{\mathrm{gas}}\times S}$$

where *T_gas_* is the gas temperature (K) and *S* represents the mass transfer surface area (m^2^), as defined in Ref. [37] However, other studies define *S* as the overall gas–liquid interfacial area of HFMs [41,45]. Typically, CO_2_ absorption flux was determined by measuring the CO_2_ concentration at the inlet and outlet of the membrane modules using chemical titration methods. However, some references (see Refs. [38,39]) did not provide details regarding the specific titration methods used or potential errors from CO_2_ measurement methods. In contrast, some authors specified the titration methods used, including potassium hydrogen phthalate [40] and sulfuric acid/sodium hydroxide [42]. Ref. [42] provided the following titration reactions for these methods:

(13) $$2\;\mathrm{NaOH}\;+H_{2}{\mathrm{SO}}_{4}\to {\;\mathrm{Na}}_{2}{\mathrm{SO}}_{4}+2{\;H}_{2}O$$

(14) $$2\;\mathrm{NaOH}\;+{\mathrm{CO}}_{2}\to {\;\mathrm{Na}}_{2}{\mathrm{CO}}_{3}+{\;H}_{2}O$$

A critical limitation identified through the collected literature is the lack of clear explanations for the relationship between CO_2_ separation performance and liquid-phase temperature [37,41,42,43,44,45]. Many studies either fail to graphically illustrate or clearly describe the relationship between CO_2_ separation performance and liquid-phase temperature, often lacking sufficient detail [37,41,42,43,44,45], or do not provide additional discussion [38,39,40]. Consequently, no empirical equations describing the temperature dependence of CO_2_ absorption capacity were found in the reviewed literature [37,38,39,40,41,42,43,44,45].

**Figure 1:**
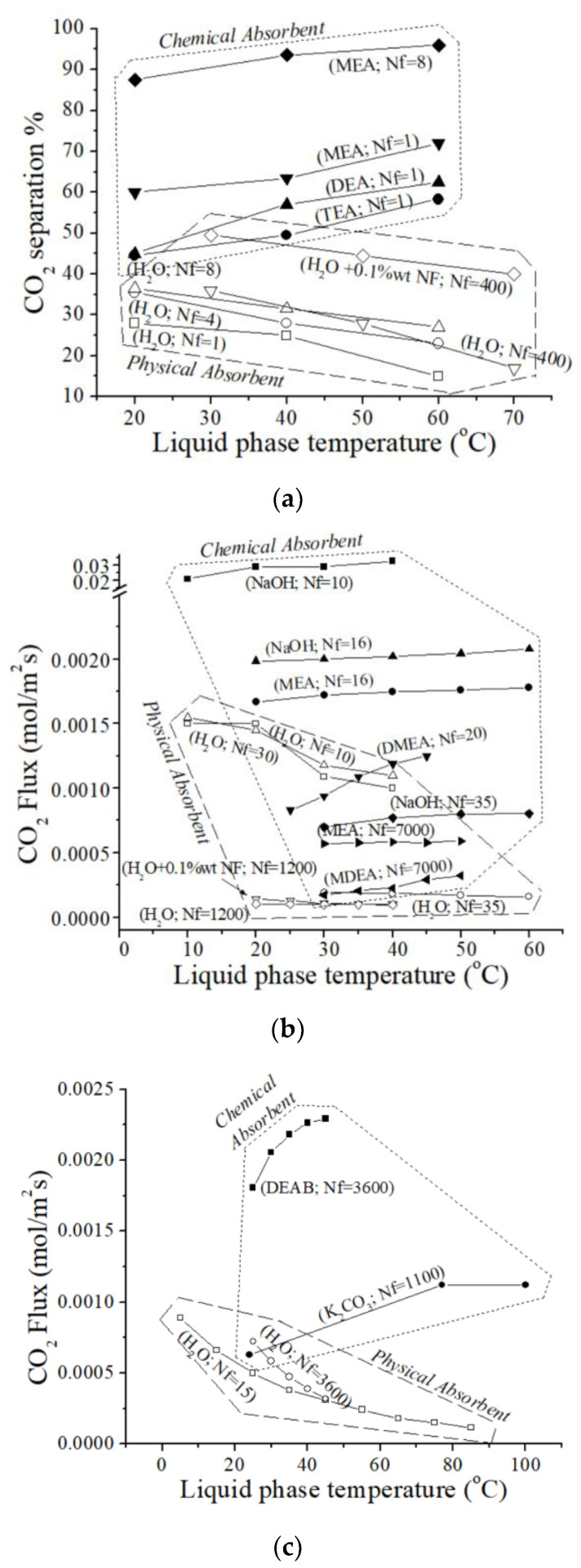
(**a**) CO_2_ separation (%) (◆, ▼, ▲, ●, △, ▽, ○, □ [37]; ▽, ◇ [41]), (**b**) CO_2_ absorption flux (mol/m^2^s) (■, △ [38]; □ [39]; ○, ◆ [40], ▽, ◇ [42]; ●, ▲ [43]; ▼ [44]; ◀, ▶ [45]), and (**c**) modeling studies (□ [21]; ● [29]; ■, ○ [46]), as a function of liquid-phase temperature.

Figure 1 graphically illustrates the relationship between temperature and CO_2_ removal performance based on data from the selected literature [21,29,37,38,39,40,41,42,43,44,45,46]. In Figure 1a, the x-axis represents temperature in degrees Celsius, and the y-axis represents the percentage of CO_2_ absorbed, as reported in the selected literature [37,41]. The numbers in parentheses following the chemical names indicate the number of polymeric fibers (Nf) used in each HFM device. Figure 1a presents data for both physical absorption with H_2_O (using HFM devices with 1 [37], 4 [37], 8 [37], and 400 [41] polymeric fibers) and chemical absorption using a variety of amines, including MEA (Nf = 1 and Nf = 8) [37], DEA (Nf = 1) [37], and TEA (Nf = 1) [37].

Analysis of the CO_2_ removal performances presented in Figure 1a (data from Refs. [37,41]) reveals two distinct trends based on the variation of liquid-phase temperature. In the first group, utilizing a physical solvent (i.e., water), CO_2_ removal performance through the microporous HFM device consistently decreases at varying rates with increasing temperature [37,41]. Conversely, the second group, characterized by chemical absorption involving a reaction between CO_2_ and the liquid phase (i.e., MEA, DEA, TEA), exhibits an increase in CO_2_ absorption capacity with increasing temperature [37]. A dotted line separates the two regions in Figure 1. As shown in Figure 1a, chemical CO_2_ removal through HFM systems generally exhibits higher CO_2_ capture performance compared to physical absorption.

Notably, the slopes of the lines representing chemical absorption vary significantly, suggesting differences in the temperature-dependent behavior of different absorbents. Additionally, a correlation was observed between the number of polymeric fibers (Nf) and CO_2_ removal performance within the single-HFM device configuration. With a single HFM, the CO_2_ capture efficiency follows the order: MEA > DEA > TEA [37]. This suggests that MEA is the most effective absorbent among these three amines for CO_2_ capture, followed by DEA and TEA. Furthermore, increasing the number of polymeric fibers (Nf) from one to eight with the same chemical absorbent (e.g., MEA) significantly improves CO_2_ capture performance [37]. A similar trend is observed for physical absorption using water, where CO_2_ separation efficiency increases with the number of polymeric fibers (Nf) from one to four and then to eight (see Figure 1a).

Based on an in-depth examination of the selected research [38,39,40,42,43,44,45], Figure 1b illustrates the complex relationship that exists between CO_2_ absorption flux and liquid-phase temperature. In particular, Figure 1b shows the CO_2_ flux (mol/m^2^s) for different absorption systems as a function of liquid-phase temperature (°C), as obtained experimentally in the literature [38,39,40,42,43,44,45]. Across all reported data, chemical absorption with NaOH [38,40,43], MEA [43,45], and DMEA [44] consistently demonstrates a higher CO_2_ removal flux compared to physical absorption with H_2_O [38,39,42]. This highlights the superior flux performance of chemical absorbents in capturing CO_2_, as already observed in Figure 1a for CO_2_ separation (%) [37,41]. The CO_2_ absorption flux for chemical absorption systems shows a marked increase with increasing liquid-phase temperature. This positive correlation suggests that elevated temperatures enhance the chemical interactions between the absorbent and CO_2_ molecules, facilitating efficient CO_2_ flux (mol/m^2^s).

In contrast to chemical absorption, the CO_2_ removal flux for physical absorption systems typically shows a slight decrease with increasing liquid-phase temperature [38,39,42]. This behavior implies that physical absorption is less sensitive to temperature changes compared to chemical absorption.

While the polymeric Nf significantly impacts CO_2_ capture performance in chemical absorption systems, its influence on physical absorption is less pronounced (see Refs. [38,39,42]). This result suggests that physical absorption is less dependent on the Nf employed in the HFM devices. When comparing chemical absorbents, NaOH appears to be better than MEA in terms of CO_2_ absorption flux, particularly with a low Nf (Nf = 16) [43]. This is further supported by comparing the flux performance achieved with Nf = 35 using NaOH [40] to that obtained with Nf = 7000 using MEA [45] within the same temperature range (30–50 °C). When comparing HFM contactors with a high Nf, such as in Ref. [45], a polymeric HFM contactor with Nf = 7000 in the presence of MEA again emerges as the more effective absorbent, exhibiting a higher CO_2_ absorption flux than MDEA.

Figure 1c presents the results of modeling studies conducted by various researchers and included in this analysis [21,29,46]. It illustrates the relationship between CO_2_ absorption flux (mol/m^2^s) and liquid-phase temperature. The thermal behavior characteristics observed in the modeling studies, including CO_2_ absorption into reactive 4-diethylamino-2-butanol solution (DEAB, Nf = 3600 [46]), K_2_CO_3_ solution (Nf = 1100 [29]), and physical absorption of CO_2_ in water (Nf = 3600 [46], and Nf = 15 [21]) through HFM devices, exhibited similar trends to those observed in the experimental studies described above.

Several key observations can be made from these results:

1. Regardless of the number of polymeric fibers (Nf) used, chemical absorption [21,46] generally exhibits higher CO_2_ capture efficiency than physical absorption [29,46], consistent with the findings presented in Figure 1a,b;
2. For chemical absorption systems, CO_2_ absorption flux typically increases significantly with increasing liquid-phase temperature, consistent with the trends observed in Figure 1a,b [29,46];
3. In contrast to chemical absorption, physical absorption generally shows a decrease in CO_2_ absorption flux as liquid-phase temperatures increase. For example, Saidi et al. [46] observed that increasing the temperature in a DEAB system from 25 °C to 45 °C led to a maximum CO_2_ flux of 8.28 mol/m^2^h. In contrast, in the same HFM device using water as the absorbent, increasing the temperature from 25 °C to 45 °C resulted in a decrease in CO_2_ absorption capacity from 2.57 to 1.27 mol/m^2^h [46]. This trend aligns with findings from other studies, which suggest that temperature has a greater influence on reaction rate than other factors limiting CO_2_ absorption, such as gas and liquid flow rates.

Overall, the trends observed in Figure 1c align with those observed in Figure 1a,b, further supporting the superior performance of chemical absorption for CO_2_ capture across a range of liquid-phase temperatures and polymeric HFM configurations. This is further supported by the contrasting temperature effects: physical absorption exhibits a decrease in CO_2_ flux with increasing temperature, while chemical absorption demonstrates a positive correlation, leading to an increased CO_2_ flux.

**Figure 2:**
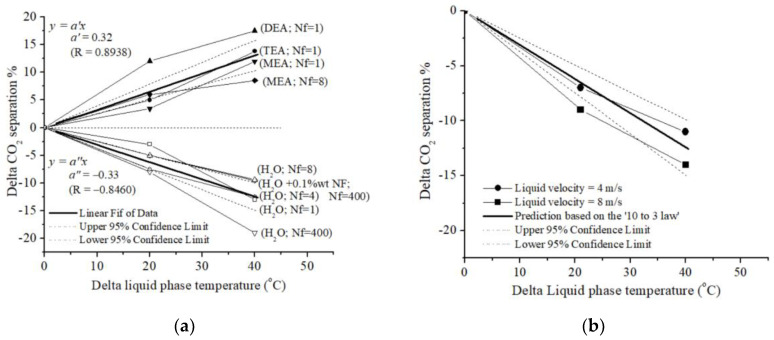
Normalized values of (**a**) CO_2_ separation (%) (◆, ▼, ▲, ●, △, ▽, ○, □ [37]; ▽, ◇ [41]), and (**b**) comparison of proposed prediction based on the empirical ’10-to-3 law’ with normalized CO_2_ separation percentages obtained from Ref. [51] (●, liquid velocity = 4 m/s; ■, liquid velocity = 8 m/s) for a PVDF HFM contactor (H_2_O).

Figure 2 presents the normalized relationships between changes in CO_2_ separation efficiency and changes in liquid-phase temperature for the solvents used in the carbon capture process. In order to improve the visual comparison of the data points’ relative differences, normalization was applied in this work. Figure 2 was created using data points from the literature (see Table 2 and Table 3) [37,38,39,40,41,42,43,44,45]. For each of 104 experimental points ($T_{\mathrm{relative}}$), the minimum value ($T^{\mathrm{minimum}\;\mathrm{value}}$) of the x-axis baseline temperature was subtracted, and the results were normalized to zero ($T_{\mathrm{normalized}}^{0^{o}C}$) as follows:

(15) $$T_{\mathrm{normalized}}^{0^{o}C}=T_{\mathrm{relative}}-{\;T}^{\mathrm{minimum}\;\mathrm{value}}$$

In this way, the x-axis of Figure 2 represents the relative ΔT of the liquid-phase temperature. This ΔT is the difference between the higher temperature of the liquid phase of the liquid solvent used in a given experiment and the liquid phase of the solvent at a lower temperature, as referred to in the literature [37,38,39,40,41,42,43,44,45]. The normalization to zero was also repeated for the performance (CO_2_ separation; y-axis) data collected from the literature [37,38,39,40,41,42,43,44,45], using the following equation:

(16) $${\mathrm{Perform}}_{\mathrm{normalized}}^{0^{o}C}={\mathrm{Perform}}_{\mathrm{relative}}-{\;\mathrm{Perform}}^{\mathrm{minimum}\;\mathrm{value}}$$

Figure 2a intuitively demonstrates that increasing temperature can lead to either an increase or a decrease in normalized CO_2_ separation performance, depending on the specific solvent. Similar to Figure 1a, Figure 2a presents 24 normalized experimental data points obtained from the literature for four different solvents: MEA [37], TEA [37], DEA [37], and H_2_O [37,41]. Data for MEA, TEA, and DEA were obtained using a single-fiber contactor (Nf = 1) [37], while additional data for MEA and H_2_O were collected using contactors with Nf ranging from 4 [37] to 400 [41].

The slopes of the individual experimental data points in Figure 2a visually represent the overall trend of CO_2_ separation performance for both physical and chemical absorption processes. In particular, Figure 2a shows an inverse relationship between normalized CO_2_ separation percentage and liquid-phase temperature for physical absorption processes. This indicates that as the temperature of the liquid phase increases, the amount of CO_2_ captured decreases. In contrast, chemical absorption processes generally exhibit an increase in performance with increasing liquid temperature.

Additionally, Figure 2a also reveals significant differences in the slopes of the normalized CO_2_ separation curves for different solvents. For example, the slopes for DEA (Nf = 1) and TEA (Nf = 1) are steeper than the slope obtained for MEA (Nf = 1) [37]. This trend persists even when compared with a higher Nf (Nf = 8) [37]. Figure 2a is also valuable for understanding the relationship between water temperature and carbon capture efficiency. As a first approximation, based on Figure 2a, it can be concluded that CO_2_ capture devices utilizing physical absorption with a high Nf (e.g., Nf = 400) exhibit a pronounced negative slope.

All data points for both physical and chemical processes were fitted to a linear regression model based on the equation presented in Figure 2a. The resulting linear regression equations are as follows:

(17) $${\mathrm{CO}}_{2}\;\mathrm{separation}\;\left(\mathrm{percentage}\right)=+0.32\;T\left({}^{\circ}C\right)$$

(18) $${\mathrm{CO}}_{2}\;\mathrm{separation}\;\left(\mathrm{percentage}\right)=-0.33\;T\left({}^{\circ}C\right)$$

Notably, the average slopes of the linear regression lines for chemical process data (upper part of Figure 2a; Equation (17)) and physical process data (lower part of Figure 2a; Equation (18)) are remarkably similar, with values of approximately +0.3% and −0.3%, respectively. This suggests that regardless of the specific process, the average trend across a large number of experiments is consistent in magnitude but opposite in direction. Within the temperature range considered, there is a statistically significant complementary relationship between temperature effects on CO_2_ separation performance for physical and chemical processes.

Assuming this trend is specific to polymeric HFM systems—and applicable to ceramic-based HFMs—it can be summarized by the ’10-to-3’ rule: for every 10 °C change in temperature, a corresponding 3% change in performance can be expected, regardless of specific conditions or other variables. For example, the ‘10 to 3’ rule means that if the liquid-phase temperature increases by 40 degrees, the CO_2_ separation percentage in a CO_2_ chemical absorption process will be approximately 12%. This empirical law could have significant predictive and economic implications for HFM systems. It suggests that the polymeric HFM systems have a limited potential for performance improvement, with a maximum of 3% change for every 10 °C temperature adjustment. This inherent constraint implies little room for substantial energy savings or efficiency gains in polymeric-based HFM CO_2_ separation processes through temperature optimization alone. Consequently, from an economic perspective, investments in temperature control for performance enhancement may yield only marginal returns, potentially impacting the cost-effectiveness of HFM technology in CO_2_ separation applications. Another aspect to consider is that in the case of a gas–liquid absorption involving chemical reactions, it typically causes significant temperature changes in the liquid phase, particularly near the gas–liquid interface of the HFM. The CO_2_ reaction and dissolution processes generate heat, which is responsible for this temperature increase [49]. For example, Pandya [50] showed that in a second-order reaction (rate proportional to the product of CO_2_ and MEA concentrations) the liquid phase leaves the adiabatic packed column at more than 10 °C higher than the inlet liquid temperature. It should be noted that this phenomenon is never considered in the examined literature [21,29,37,38,39,40,41,42,43,44,45,46] nor in other studies on “isothermal” experiments for the CO_2_ chemical absorption process.

The empirical ’10-to-3’ rule was validated using simulation results from Jalali et al. [51] for physical CO_2_ removal in distilled water. In this section, we compare the results of the proposed easy rule with simulation data obtained from process optimization of CO_2_ removal using a PVDF-HFM system. These simulations were conducted using two-dimensional (2D) computational fluid dynamics (CFD) based on mass transfer equations for the HFM contactor (fiber outer diameter = 0.95 mm; fiber inner diameter = 0.60 mm; length = 150 mm; Nf = 30). The simulations assumed steady-state and laminar conditions and employed a finite-element approach [51]. Figure 2b presents the normalized CO_2_ separation percentages obtained from Ref. [51] for a PVDF HFM system at various H_2_O temperatures (5 °C, 26 °C, and 45 °C) and liquid velocities (4 m/s and 8 m/s). The results demonstrate that H_2_O temperature influences the convective mass transfer in the polymeric HFM. This process, described by the continuity equation, in turn affects the overall CO_2_ mass transfer. Figure 2b shows that the predictions based on the ’10-to-3’ rule align well with the simulation results across different liquid velocities for physical CO_2_ removal in distilled water.

**Figure 3:**
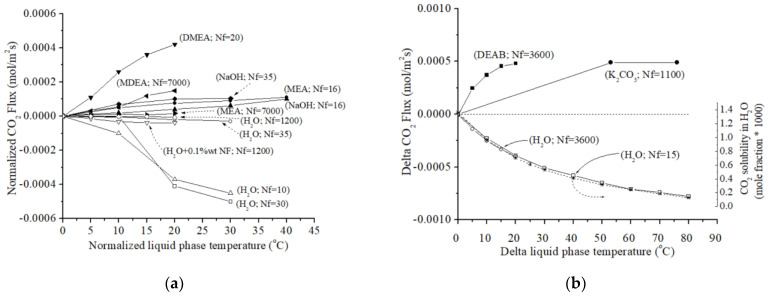
Normalized values of (**a**) CO_2_ absorption flux (mol/m^2^s) (△ [38]; □ [39]; ○, ◆ [40], ▽, ◇ [42]; ●, ▲ [43]; ▼ [44]; ◀, ▶ [45]) and (**b**) comparison of modeling studies (□ [21]; ● [29]; ■, ○ [46]) with experimental data on CO_2_ solubility in water [18] (*). The left *y*-axis represents CO_2_ absorption flux (mol/m^2^s) [21,29,46], while the right *y*-axis shows CO_2_ solubility in water (see Table 1) [18].

Figure 3a presents a graph illustrating the relationship between normalized liquid-phase temperature and capture performance. The capture performance is expressed as CO_2_ flux (mol/m^2^s) obtained from a large number of experiments, as previously explained [38,39,40,42,43,44,45]. Similar to Figure 2, here delta liquid-phase temperature represents the difference between the higher and lower liquid-phase temperatures, while delta CO_2_ flux quantifies the change in CO_2_ flux associated with this temperature difference. Figure 3(a) presents detailed data for more than sixty experiments, standardized in this current analysis, using various absorbent types: DMEA [44], MDEA [45], NaOH [40,43], MEA [43,45], and H_2_O [38,39,40,42]. In addition, Figure 3a presents normalized data for different HFM contactors with varying Nf: 10 [38], 16 [43], 20 [44], 30 [39], 35 [40], 7000 [45], and 1200 [42]. All the selected experimental results investigated CO_2_ flux performance across a temperature range of 40 °C.

Some observations can be noted from Figure 3a. First, the figure shows that, in general, the normalized CO_2_ flux increases only slightly with increasing liquid-phase temperature in chemical absorption processes. Contrary to initial expectations, elevating the temperature does not significantly improve CO_2_ capture efficiency compared to operation at ambient temperatures. However, DMEA [44] appears to be an exception, exhibiting potentially better performance at elevated temperatures. Except for the cases described in Refs. [38,39], the performance of physical absorbers using water is minimally affected by relatively small increases in water temperature.

Figure 3a demonstrates that, from a graphical perspective, a high number of polymeric fibers (Nf) does not necessarily correspond to a large variation in performance for either chemical or physical absorption processes. Conversely, devices with a lower number of polymeric fibers seem to be more sensitive to temperature changes, regardless of the absorbent type. Interestingly, with a few exceptions—see Nf = 10 (H_2_O) [38], Nf = 20 (DMEA) [44], and Nf = 30 (H_2_O) [39]—HFM systems with a lower number of fibers exhibit a greater variation in performance with increasing temperature compared to systems with a larger number of fibers, e.g., Nf = 7000 (MEA-MDEA) [45] and Nf = 1200 (H_2_O) [42]. The overall change in liquid temperature has been confirmed to influence CO_2_ flux performance (either positively or negatively). However, this effect is less pronounced when considering the data as a whole.

The above observation might be linked to the interaction between fiber dimensions and temperature. For instance, Refs. [43,45] employ HFM contactors with significantly different fiber sizes. As an example, Ref. [43] describes an HFM contactor with 16 fibers (Nf = 16; NaOH), where the outer diameter (F.Od) and inner diameter (F.Id) are 1.1 mm and 0.42 mm, respectively. Conversely, the second HFM system [45] utilizes a significantly larger Nf (Nf = 7000; MEA-MDEA) with dimensions of 0.442 mm and 0.344 mm for F.Od and F.Id, respectively. This observation appears to be linked not only to the Nf but also to the overall length of the HFM device. To illustrate this point, consider an HFM contactor composed of three modules connected in series [52]. Each module is 20 cm long, resulting in a total length of 60 cm. These modules contain 20 PTFE HFMs with an F.Od of 1.7 mm and an F.Id of 1.0 mm. In this specific configuration, the relative temperature variation from 25 °C to 45 °C for the 3-diethylaminopropylamine (DEAPA) absorbent at two different concentrations (1 and 2 mol/L) is negligible compared to the effects observed in selected studies [40,43,44,45]. This is further supported by the performance results obtained by Chen et al. [52], which are presented in Figure S2 of the Supplementary Materials.

In addition, the fact that a polymeric HFM system with relatively few fibers exhibits performance similar to a more complex device with a very high Nf is confirmed in the work of Luqmani et al. [53]. The membrane devices comprising 10,200 microporous PP HFMs (fiber inert diameter = 240 μm; fiber wall thickness = 30 μm; length= 0.16 m) for a physical absorption process in H_2_O [53] showed the same qualitative and quantitative behavior of an HFM contactor with relatively few fibers [38,39]. Figure S3 in the Supplementary Materials shows the direct comparison between an HFM contactor studied by Luqmani et al. [53] with Nf = 10200 (H_2_O; liquid-phase velocity = 1.5 × 10^−3^ and 2.5 × 10^−3^ m/s) and two other HFM contactors with Nf = 10 (H_2_O) [38] and Nf = 30 (H_2_O) [39], respectively. Thus, from this direct comparison between two completely different HFM systems—one with Nf = 10,200 [53] and the other two with fewer fibers [38,39]—the final CO_2_ flux exhibits the same behavior with respect to temperature.

Figure 3b compares the normalized values of CO_2_ absorption flux (mol/m^2^s) obtained from various conventional split-flow absorber processes [46] and mathematical modeling studies [21,29] for different absorbents: DEAB (Nf = 3600 [46]), K_2_CO_3_ solution (Nf = 1100 [29]), and H_2_O (Nf = 3600 [46], and Nf = 15 [21]). For comparison, the x-axis also presents the solubility of CO_2_ in water (see Table 1) from Ref. [18]. The figure highlights key findings regarding the relationship between liquid temperature and CO_2_ removal performance.

For chemical absorption by reactive DEAB (Nf = 3600 [46]) and K_2_CO_3_ solution (Nf = 1100 [29]), Figure 3b shows two distinct curves that converge towards a common horizontal asymptote. Notably, DEAB reaches its maximum performance within a relatively narrow temperature range of approximately 20 °C, while K_2_CO_3_ requires a significantly larger temperature difference (approximately 80 °C) to achieve its peak performance. From an energy perspective, heating the DEAB absorber is generally more energy-efficient than heating the K_2_CO_3_ absorber.

Furthermore, the data suggest a significant decrease in CO_2_ absorption flux with increasing normalized temperature of H_2_O across the modeling studies [21,46]. This trend can be readily explained by the dependence of CO_2_ solubility on absorption flux. As liquid-phase temperature increases, the solubility of CO_2_ in water generally decreases (as shown on the left-side axes of Figure 3b). This decrease in solubility can limit the amount of absorbed CO_2_ in water and potentially contribute to the observed variations in CO_2_ absorption flux across the modeling studies, irrespective of other parameters. Against our expectations, the results obtained from different mathematical models [21,46] show excellent agreement with the well-known data on CO_2_ solubility in water, as reported in [18] (see Table 1).

In conclusion, CO_2_ gas mixtures are frequently produced by several industrial processes, including the production of biogas, coal gasification, and steam methane reforming [54,55]. By investigating the complex effect of liquid-phase temperature—a variable that has been relatively neglected in previous studies—in various gas–liquid polymeric HFM contactors, this work aimed to optimize CO_2_ separation. The results presented here fill the knowledge gap on polymeric HFM contactor operation for CO_2_ removal.

## 5. Conclusions

Efficient CO_2_ removal is indispensable for the successful implementation of various technologies. The present analysis aimed to investigate the overall effects of liquid-phase temperature on CO_2_ removal performance across several physical and chemical absorption processes utilizing polymeric HFM contactors. Overall, the findings of this study demonstrate the impact of liquid-phase temperature on CO_2_ separation performance.

Our work confirms that CO_2_ absorption flux consistently increases with increasing liquid-phase temperature in chemical absorption processes, while it consistently decreases with increasing temperature in physical absorption processes using water. Several key conclusions were drawn:

1. Superiority of Chemical Absorption: CO_2_ chemical absorption consistently outperforms physical absorption using H_2_O as the absorbent;
2. Temperature-Dependent Enhancement: CO_2_ chemical absorption flux exhibits a positive correlation with temperature, indicating enhanced performance at higher temperatures;
3. Inverse Relationship with Temperature: In contrast, CO_2_ removal capacity in H_2_O decreases with increasing temperature.

Furthermore, we have identified quantitative relationships between CO_2_ separation (%) and temperature for both chemical and physical absorption processes:

- Chemical absorption → CO_2_ separation (%) = +0.3 T (°C);
- Physical absorption → CO_2_ separation (%) = −0.3 T (°C).

Statistical analysis strongly supports these trends, with high R-values (>0.8) observed for both chemical and physical absorption processes. In addition, these two empirical laws were confirmed by direct comparisons with additional experimental results.

These considerations provide valuable tools for predicting CO_2_ separation performance under varying temperature conditions. Quantitatively, the equations predict a 3% increase in CO_2_ separation per 10 °C temperature rise for chemical absorption, while a 3% decrease is predicted for physical absorption. It is crucial to acknowledge that these equations provide rough predictions based on simplified models and may not be universally applicable.

Given the substantial volume of data analyzed, these findings are statistically significant and provide robust insights into the temperature–performance relationships and inherent limitations of these absorption processes. Actual CO_2_ separation values may deviate from these predicted values due to various factors, including operating conditions (e.g., gas composition, pressure), absorbent properties (e.g., type, concentration, flow rate), and membrane characteristics (e.g., HFM thickness, number of polymeric fibers, inner module radius, length, porosity, tortuosity). Furthermore, the presence of impurities and potential limitations in HFM stability can also influence performance.

## 6. Prospects

While CO_2_ capture utilizing polymeric HFM contactors has garnered significant interest in recent years, with promising results reported [6,8,9,10,11,56], a more comprehensive understanding of how liquid-phase temperature impacts both physical and chemical CO_2_ removal processes remains crucial. This section explores potential future research directions to further investigate gas–liquid polymeric HFM systems.

### 6.1. Economic Optimization of Liquid-Phase Temperature

A key area for future research is to determine the optimal liquid-phase temperature from an economic perspective. This involves understanding the trade-offs between:

- Decreased efficiency in physical absorption due to the reduced solubility of CO_2_ at lower temperatures;
- Enhanced efficiency in chemical absorption due to increased reaction kinetics at higher temperatures;
- Energy costs associated with liquid-phase temperature control;
- Energy costs associated with physical solvent regeneration (e.g., through temperature swing, pressure swing, or stripping with air or steam);
- Energy costs associated with chemical solvent regeneration via the introduction of heat to regenerate the original chemical solvent and obtain pure CO_2_.

### 6.2. High-Pressure Liquid-Phase Studies

New detailed studies on the performance of HFM contactors under high liquid-phase pressure are crucial. Following some interesting results reported by Dindore et al. [57] with PP HFM, there is currently a lack of research on the performance of polymeric HFM devices operating under very high liquid-side pressures. Theoretically, increasing liquid-phase pressure can benefit both absorption processes:

- Physical absorption: Higher liquid-phase pressure enhances the solubility of CO_2_ in the liquid absorbent (i.e., H_2_O) [18,20], leading to improved absorption capacity;
- Chemical absorption: Elevated liquid-phase pressure can increase the density of the liquid, potentially enhancing mass transfer and CO_2_ chemical absorption [38].

To assess the applicability of high liquid pressure, it is crucial to investigate its impact on polymeric membrane wetting over extended operation periods. Furthermore, exploring the influence of correspondingly high pressure on the gas side is important to assess its potential role in mitigating wetting or affecting other performance aspects.

### 6.3. Low- and High-Temperature Liquid-Phase Studies

Future research should explore the development of new physical solvents suitable for chilled CO_2_ capture processes, specifically those capable of operating under ambient or sub-zero temperatures (−100 °C < T < 25 °C). Thermally stable chemical solvents suitable for high-temperature industrial processes can enhance CO_2_ capture and reduce regeneration energy requirements by narrowing the temperature gap between capture and regeneration.

### 6.4. Long-Term Stability Tests and Polymeric Nature

While the selected literature focuses on the effect of liquid-phase temperature on physical and chemical CO_2_ absorption using well-known polymeric HFM devices, significant gaps remain in our understanding of long-term stability and the behavior of novel polymeric HFM materials under varying liquid-phase temperatures. Here are some key areas for further investigation:

- Long-Term Stability Tests: Current research focuses on CO_2_ capture within a limited temperature range of the liquid phase. However, industrial applications may necessitate operation at higher or lower temperatures. Long-term stability tests at different liquid-phase temperatures are crucial to evaluate the long-term performance of traditional polymeric HFM contactors under these more demanding conditions.
- Membrane Wetting: Future studies should more explicitly investigate the potential for polymeric HFM wetting under varying operating conditions. This includes a systematic evaluation of the impact of elevated temperatures and the use of hydrophilic solvents on the long-term stability and performance of polymeric HFMs, as wetting can significantly hinder their efficiency.
- Advanced HFM Materials: Expanding the investigation beyond polymeric HFMs is essential. New materials, such as advanced ceramics, thin-film composites, facilitated transport membranes, and mixed-matrix membranes, offer potential advantages like enhanced durability and improved selectivity, potentially even at temperatures other than room temperature.

## Figures and Tables

**Table 1:** Solubility of CO_2_ in water at atmospheric pressure (solubility in mole fraction of CO_2_ in the liquid phase × 1000) [18].

T (°C)	0	10	20	30	40	50	60	70	80	90
Solubility	1.362	0.962	0.704	0.531	0.409	0.319	0.247	0.185	0.127	0.067

**Table 2:** Physical CO_2_ capture using polymeric HFM contactors [37,38,39,40,41,42]. Data organized and arranged using the number of polymeric fibers (Nf) as the primary classification criterion.

Ref.	[37]	[37]	[37]	[38]	[39]	[40]	[41]	[42]	[42]
Fiber	PTFE	PTFE	PTFE	PVDF	PVDF	PVDF	PP	PP	PP
Liquid	H_2_O	H_2_O	H_2_O	H_2_O	H_2_O	H_2_O	H_2_O NF	H_2_O NF	H_2_O NF
Ti (°C)	20	20	20	10	10	30	30	20	20
Tf (°C)	60	60	60	40	40	60	70	40	40
Nf	1	4	8	10	30	35	400	1200	1200
F.Od (mm)	3.52	1.84	1.2	1	0.95	1	0.45	0.4	0.4
F.Id (mm)	2.96	1.46	0.68	0.55	0.6	0.65	0.32	0.3	0.3
F.l (mm)				150	150		400	250	250
M.Pd (µm)	0.51	0.58	0.48			0.2	0.15	0.2	0.2
M.P (%)	52	52	52		77.1	0.75 *		50	50
B.p (psi)	12.3	14	14.8						
M.Od (mm)	6.35	6.35	6.35						
M.Id (mm)	4.78	4.78	4.78	14	14	10	20	34	34
M.l (mm)	230	230	230	270	270	270			
C.A. (cm^2^)	42	88	115			190	1600		
P.D.	54	59	51	204	582				

PTFE = polytetrafluoroethylene, PVDF = polyvinylidene fluoride, PP = polypropylene, NF = nanofluid, Ti = initial temperature, Tf = final temperature, Nf = number of fibers, F.Od = fiber outer diameter, F.Id = fiber inert diameter, F.l = fiber length, M.Pd = membrane pore diameter, M.P = membrane porosity, B.p = bubble point, M.Od = module outer diameter, M.Id = module inert diameter, M.l = module length, C.A. = contact area, P.D. = packing density in % [37] or m^2^/m^3^ [38,39]; * = no units (omitted by the authors).

**Table 3:** Chemical CO_2_ capture using polymeric HFM contactors [37,38,40,43,44,45]. Data organized and arranged using the number of polymeric fibers (Nf) as the primary classification criterion.

Ref.	[37]	[37]	[37]	[37]	[38]	[43]	[43]	[44]	[40]	[45]	[45]
Fiber	PTFE	PTFE	PTFE	PTFE	PVDF	PVDF	PVDF	PTFE	PVDF	PP	PP
Liquid	TEA	DEA	MEA	MEA	NaOH	MEA	NaOH	DMEA	NaOH	MDEA	MEA
Ti (°C)	20	20	20	20	10	20	20	25	30	30	30
Tf (°C)	60	60	60	60	40	60	60	44	60	50	50
Nf	1	1	1	8	10	16	16	20	35	7000	7000
F.Od (mm)	3.52	3.52	3.52	3.52	1	1.1	1.1	1.7	1	0.442	0.442
F.Id (mm)	2.96	2.96	2.96	2.96	0.55	0.42	0.42	1	0.65	0.344	0.344
F.l (mm)					150					800	800
M.Pd (µm)	0.51	0.51	0.51	0.5							
M.P (%)	52	52	52	52				50	0.75 *	>45	>45
B.p (psi)	12.3	12.3	12.3	12.3							
M.Od (mm)	6.35	6.35	6.35	6.35		15	15				
M.Id (mm)	4.78	4.78	4.78	4.78	14	11	11	1.8	10	80	80
M.l (mm)	230	230	230	230	270	160	160	200	270	1000	1000
C.A. (cm^2^)	42	42	42	42		34	34		190	60,500	60,500
P.D.	54	54	54	54	204	17 *	17 *			21.4	21.4

PTFE = polytetrafluoroethylene, PVDF = polyvinylidene fluoride, PP = polypropylene, NF = nanofluid, Ti = initial temperature, Tf = final temperature, Nf = number of fibers, F.Od = fiber outer diameter, F.Id = fiber inert diameter, F.l = fiber length, M.Pd = membrane pore diameter, M.P = membrane porosity, B.p = bubble point, M.Od = module outer diameter, M.Id = module inert diameter, M.l = module length, C.A. = contact area, P.D. = packing density in % [37] or m^2^/m^3^ [38,39]; * = no units (omitted by the authors).

## Data Availability

The raw data supporting the conclusions of this article will be made available by the authors upon request.

## Supplementary Materials

The following supporting information can be downloaded at https://www.mdpi.com/article/10.3390/polym17101387/s1, Figure S1: Liquid-phase temperature ranges studied in the selected literature for (a) physical CO_2_ capture as reported in Table 2 [37,38,39,40,41,42] in the manuscript, and (b) chemical CO_2_ capture (see Table 3) [37,38,40,43,44,45] using polymeric HFM contactors [37,38,39,40,41,42,43,44,45]. The figures show the location of all the literature data points used in this study [37,38,39,40,41,42,43,44,45]; Figure S2: Impact of normalized liquid-phase temperature on normalized CO_2_ flux (mol/m^2^s) for 3-diethylaminopropylamine (DEAPA) at two different concentrations (1 and 2 mol/L) across a microporous PTFE HFM contactor composed of three modules connected in series [52]. Each module is 20 cm long, resulting in a total length of 60 cm; Figure S3: Impact of normalized liquid-phase temperature (H_2_O) on normalized CO_2_ flux (mol/m^2^s) across a microporous (PP) HFM contactor recently studied by Luqmani et al. [53] with Nf = 10,200, and another two HFM contactors with Nf = 10 (H_2_O) [38] and Nf = 30 (H_2_O) [39], respectively.
