# Supplementary material for: Polymeric Membrane Contactors for CO2 Separation: A Systematic Literature Analysis of the Impact of Absorbent Temperature
Source: Polymers (Basel). 2025 May 18;17(10):1387. doi: 10.3390/polym17101387 (PMC12115163; doi:10.3390/polym17101387)
Supplement: Supplementary file 1 [file polymers-17-01387-s001.zip › polymers-3629057-supplementary.pdf]

Article

Polymeric Membrane Contactors for CO_2 Separation: A Systematic Literature Analysis of the Impact of Absorbent Temperature

Edoardo Magnone, Min Chang Shin and Jung Hoon Park *

Department of Chemistry & Biochemical Engineering, Dongguk University, Manhae gwan, Room E629, 30, Pildong-ro 1gil, Jung-gu, Seoul 100-715, Republic of Korea; magnone.edoardo.korea@gmail.com (E.M.); gogokill31@naver.com (M.C.S.)

* Correspondence: pjhoon@dongguk.edu; Tel.: +82-2-2260-8598

Supplementary material

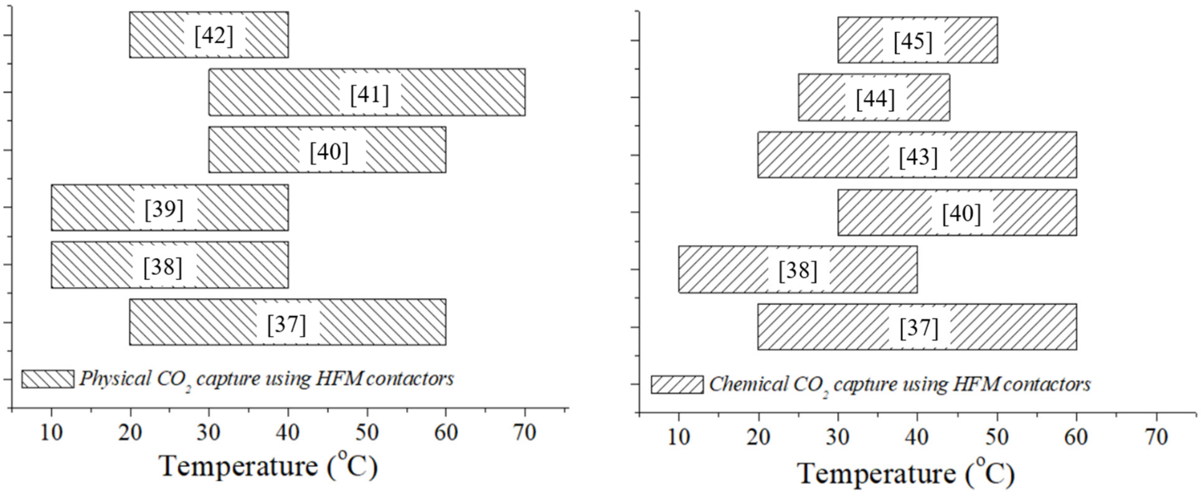

Figure S1. Liquid-phase temperature ranges studied in the selected literature for (a) physical CO_2 capture regarding Table 2 [37–42] in the manuscript, and (b) chemical CO_2 capture (see Table 3) [37,38,40,43–45] using polymeric HFM contactors [37-45]. The figures show the location of all the literature data points used in this study [37-45].

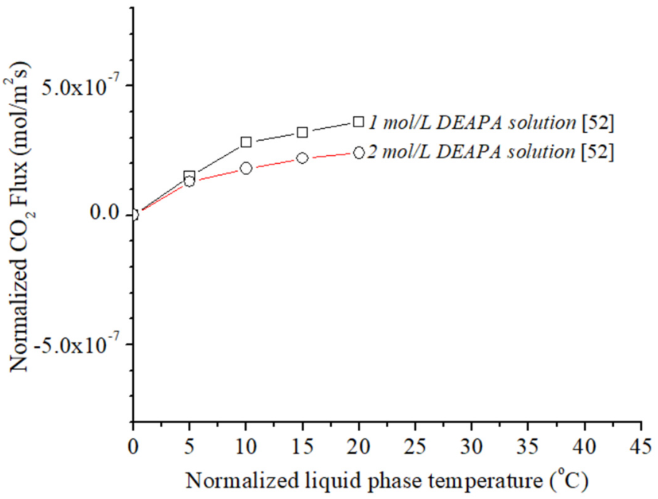

Figure S2. Impact of normalized liquid phase temperature on normalized CO_2 flux (mol/m^2s) for 3-diethylaminopropylamine (DEAPA) at two different concentrations (1 and 2 mol/L) across a microporous PTFE HFM contactor composed of three modules connected in series [52]. Each module is 20 cm long, resulting in a total length of 60 cm.

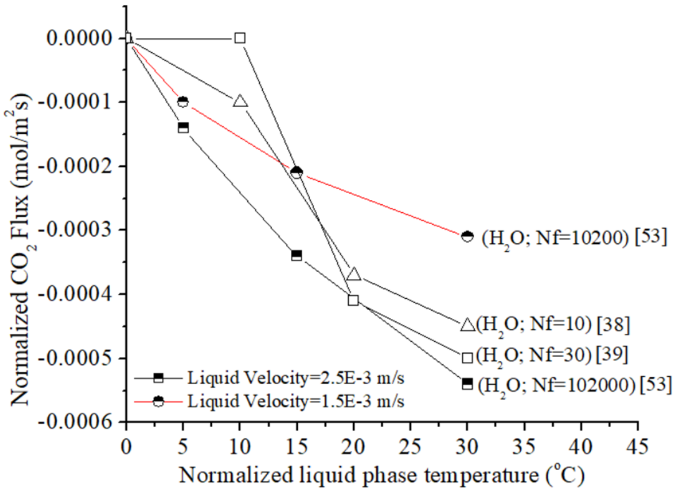

Figure S3. Impact of normalized liquid phase temperature (H20) on normalized CO_2 flux (mol/m^2s) across microporous (PP) HFM contactor recently studied by Luqmani et al. [53] with Nf = 10200 and another two HFM contactors with Nf = 10 (H_2O) [38] and Nf = 30 (H_2O) [39], respectively.
